# Long-Term Outcome of Anti-Glomerular Basement Membrane Antibody Disease Treated with Immunoadsorption

**DOI:** 10.1371/journal.pone.0103568

**Published:** 2014-07-31

**Authors:** Peter Biesenbach, Renate Kain, Kurt Derfler, Thomas Perkmann, Afschin Soleiman, Alexandra Benharkou, Wilfred Druml, Andrew Rees, Marcus D. Säemann

**Affiliations:** 1 Internal Medicine III/Clinical Division of Nephrology & Dialysis, Medical University of Vienna, Vienna, Austria; 2 Clinical Institute of Pathology, Medical University of Vienna, Vienna, Austria; 3 Department of Laboratory Medicine, Medical University of Vienna, Vienna, Austria; National Center for Scientific Research Demokritos, Greece

## Abstract

**Background:**

Anti-glomerular basement membrane (GBM) antibody disease may lead to acute crescentic glomerulonephritis with poor renal prognosis. Current therapy favours plasma exchange (PE) for removal of pathogenic antibodies. Immunoadsorption (IAS) is superior to PE regarding efficiency of antibody-removal and safety. Apart from anecdotal data, there is no systemic analysis of the long-term effects of IAS on anti-GBM-disease and antibody kinetics.

**Objective:**

To examine the long-term effect of high-frequency IAS combined with standard immunosuppression on patient and renal survival in patients with anti-GBM-disease and to quantify antibody removal and kinetics through IAS.

**Design:**

Retrospective review of patients treated with IAS for anti-GBM-antibody disease confirmed by biopsy and/or anti-GBM-antibodies.

**Setting:**

University Hospital of Vienna, Austria.

**Participants:**

10 patients with anti-GBM-disease treated with IAS.

**Measurements:**

Patient and renal survival, renal histology, anti-GBM-antibodies.

**Results:**

Anti-GBM-antibodies were reduced by the first 9 IAS treatments (mean number of 23) to negative levels in all patients. Renal survival was 40% at diagnosis, 70% after the end of IAS, 63% after one year and 50% at the end of observation (mean 84 months, range 9 to 186). Dialysis dependency was successfully reversed in three of six patients. Patient survival was 90% at the end of observation.

**Conclusion:**

IAS efficiently eliminates anti-GBM-antibodies suggesting non-inferiority to PE with regard to renal and patient survival. Hence IAS should be considered as a valuable treatment option for anti-GBM-disease, especially in patients presenting with a high percentage of crescents and dialysis dependency due to an unusual high proportion of responders.

## Introduction

Anti-glomerular basement membrane (GBM) disease is defined by circulating autoantibodies specific for the alpha-3 chain of type IV collagen [1] and characterised by focal necrotizing glomerulonephritis with linear deposition of IgG along the GBM. Those affected present with acute renal failure often accompanied by pulmonary haemorrhage because the anti-GBM-antibodies also bind to the alveolar basement membrane. Although rare with an incidence of two per million population per year, anti-GBM-disease accounts for approximately 10 to 20 percent of crescentic nephritis [2]–[4]. Untreated, it rapidly destroys the kidney emphasizing the need for rapid diagnosis and therapy [2], [5], [6].

The treatment aims at limiting further renal injury by rapidly reducing circulating anti-GBM-antibodies. The current standard treatment consists of plasma exchange (PE) combined with cyclophosphamide and corticosteroids. Although never submitted to randomised controlled clinical trials, there is compelling evidence that morbidity and mortality have improved markedly since PE was introduced [2], [6]–[11].

Immunoadsorption (IAS) with high-affinity matrices selectively binding human IgG and IgM provides an alternative way of removing antibodies and immune complexes [12]. Theoretically it is more efficient than PE because unlimited volumes of plasma can be processed at each treatment [13] whereas PE is usually restricted to a single plasma volume, resulting in higher antibody reduction of more than 85% in IAS [14] compared to up to 70% in PE [15]. IAS is successfully employed in sensitized allograft recipients and in autoimmune disorders including hemophilia A, pemphigus, thrombocytopenic purpura and lupus [13], [16]–[20], whereas only isolated case reports describe IAS against anti-GBM-disease [21]–[23], including a dialysis-dependant patient who recovered renal function despite 100% crescents in the renal biopsy [24]. Here we describe our results of high intensity IAS in 10 consecutive patients with anti-GBM-disease. Examining the effectiveness, safety and costs of IAS in anti-GBM-disease, we show that IAS is at least as effective as PE and may control anti-GBM-antibodies more rapidly.

## Patients, Materials and Methods

### Patients

The study group consisted of patients presenting with anti-GBM-disease to the University Hospital of Vienna, Austria, between 1997 and 2012. Diagnosis was based on the presence of anti-GBM-antibodies in serum by indirect immunofluorescence and ELISA (mean concentration at start of treatment 73.6 U/ml: range 6.6–207.1), and in glomeruli in patients with renal biopsies. The mean age was 29 years and five were male and five female. Eight of the patients displayed pulmonary haemorrhage. Patient details are described in the individual case reports and are summarized (Table 1).

**Table 1:** Baseline characteristics of patients treated.

			Renalfunction	Glomerula in biopsy,n (%)			Lung	
	Age (years)	Sex(male)	serum-creatinine	withcrescents		withnecrosis	confirmed inHR-CT	Follow-up (months)
Pat 1	44	No	Dialysis	3/4 (75)		0/4 (0)	None	144
Pat 2	19	No	Dialysis	25/25 (100)		19/25 (76)	Confirmed	186
Pat 3	35	Yes	1.82 mg/dl	2/7 (28)		2/7 (28)	Confirmed	5
Pat 4	19	Yes	0.96 mg/dl	No biopsy			Confirmed	49
Pat 5	18	No	1.07 mg/dl	No biopsy			Confirmed	123
Pat 6	25	No	3.7 mg/dl	5/27 (19)	2/27 (7)		Confirmed	153
Pat 7	19	Yes	Dialysis	27/27 (100)	21/27 (78)		Confirmed	51
Pat 8	25	Yes	Dialysis	24/25 (96)	9/25 (36)		Confirmed	49
Pat 9	19	Yes	Dialysis	15/16 (94)	8/16 (50)		None	72
Pat 10	66	No	Dialysis	5/8 (63)	2/8 (25)		None	9

### Treatment

Immunosuppressive regimen (Table 2) consisted of pulse corticosteroids followed by tapering doses of oral prednisolone and cyclophosphamide, given either as intravenous boli or daily oral doses of 2–3 mg/kg body weight. Additionally, all patients received a course of high intensity IAS whose frequency and duration was determined by clinical course and anti-GBM-antibody levels. Five patients were converted to IAS after 2 to 4 PE treatments after anti-GBM-antibody levels had remained refractory. IAS was performed using our standard previously published protocol [14], [19], [25]. Up to 80 ml/min of blood was taken from a central or peripheral vein and anti-coagulated with citrate and/or heparin before separation on a plasmaseparator (COBE Spectra Apheresis system, Terumo). The plasma fraction was forwarded into the Adsorption-Desorption-Automated system (ADAsorb, Medicap Ulrichstadt, Germany) prepared with either TheraSorb (Milteny Biotec, Bergisch Gladbach, Germany) or Immunosorba (Fresenius Medical Care, Bad Homburg, Germany) adsorbers selectively binding IgG, IgM and immune complexes in human plasma; the procedure was continued until 2.5 to 3 times the plasma volume had been processed.

**Table 2:** Extracorporal treatment and concomitant immunosuppression.

Immunoadsorption			Plasmaexchange	Cyclophosphamide		Steroids
# of treatments	Duration	volume	# of treatments	Oral	Pulse	
22.7±12.1	85±112 days	7660 ml	1.5±1.7	60%	60%	100%

### Clinical assessment and laboratory studies

Clinical data were recorded at diagnosis and then monthly for six months and yearly thereafter. Anti-GBM-antibodies were re-assayed in all sera using a commercial ELISA (Wieslab, Malmö, Sweden) according to the manufacturer’s instructions. Sera were batched with those from an individual patient run of the same ELISA.

### Ethics statement

The study was approved by the ethics committee of the Medical University of Vienna (EK1819/2013). Participants did not provide written or verbal consent as the retrospective design did not allow obtaining consent. Patient records were anonymized prior to analysis.

## Results

### Case reports

**Patient 1** presented with a serum-creatinine of 7.4 mg/dl necessitating hemodialysis. Anti-GBM-antibody titer was 1∶80 and renal biopsy showed necrotising glomerulonephritis with 75% crescents and linear deposition of IgG. She was treated with three dexamethasone pulses (50 mg) and prednisolone for six months. Oral cyclophosphamide was given for two months. After two PEs and refractory anti-GBM-antibodies, she was converted to IAS and received 12 sessions over 27 days resulting in negative anti-GBM-antibodies after 2 treatments but renal function did not recover. Four years later, she received a renal allograft and remains well with excellent graft function and without recurrence of anti-GBM-disease.

**Patient 2** presented anuric with hemoptysis and a serum-creatinine of 5.5 mg/dl necessitating dialysis. Renal biopsy showed necrotizing glomerulonephritis with 100% crescents and linear IgG deposition. Anti-GBM-antibody concentration was 43.8 U/ml. She was treated with six boli of cyclophosphamide (total dose of 6 g) and methylprednisolone (650 mg for three days, subsequently tapered). IAS was performed 25 times over 171 days and assays for anti-GBM-antibodies were negative after 4 weeks. Renal function recovered and she was discharged with a serum-creatinine of 2 mg/dl. After three years maintenance immunosuppression (mycophenolate mofetil 1 g/day and prednisolone 5 mg/day) was stopped while serum-creatinine was 2.5 mg/dl. However, her renal function deteriorated despite negative anti-GBM-antibodies testing. Renal biopsy showed chronic scarring without active glomerulonephritis or linear deposition of IgG. She restarted dialysis 64 months after diagnosis and received four years later a renal transplant with excellent graft function and no recurrence of anti-GBM-disease. The initial course of the patient has previously been reported [24].

**Patient 3** presented with macroscopic hematuria, a serum-creatinine of 1.8 mg/dl and proteinuria (5.5 g/day) without pulmonary involvement. Anti-GBM-antibody concentration was 24.5 U/ml. Renal biopsy showed necrotizing glomerulonephritis with 28% crescents and linear deposition of IgG. He was treated with three 250 mg pulses of prednisolone (followed by 3 months of oral prednisolone), cyclophosphamide (150 mg daily) and IAS, 16 sessions over four weeks. After 1 week anti-GBM-antibodies tested negative and serum-creatinine initially stabilized at 1.75 mg/dl before rising to 3.8 mg/dl after three months despite continuing immunosuppression. He developed severe hemoptysis and a second renal biopsy revealed necrotising glomerulonephritis with fibrocellular crescents in two thirds of glomeruli. Three further pulses of methylprednisolone (250 mg) were given and he received ten PE sessions but did not respond. IAS was re-instituted for 23 sessions together with empiric intravenous immunoglobulin therapy. Renal function stabilized but after two weeks the patient was readmitted with sepsis and died three weeks later with multi-organ failure.

**Patient 4** presented with haemoptysis and respiratory distress together with macroscopic haematuria and proteinuria (5 g/day): serum-creatinine was normal and anti-GBM-antibody titer was 1∶160. The patient was treated with three daily pulses of methylprednisolone, intravenous cyclophosphamide (5 times 200 mg) and IAS. Symptoms resolved and anti-GBM-antibodies became undetectable but IAS was continued at lower intensity (45 treatments over one year) because treatment with cyclophosphamide had to be stopped due to anaemia. Anti-GBM-antibodies remained undetectable and proteinuria returned to normal being stable for four years along with serum-creatinine.

**Patient 5** presented with respiratory distress, macroscopic hematuria and proteinuria (0.6 g/day) and an initial serum-creatinine of 1.21 mg/dl. Anti-GBM-antibody titer was 1∶80 and she was treated with prednisolone (50 mg daily) and 18 sessions of IAS over 10 weeks. The symptoms resolved and the serum-creatinine fell to 0.8 mg/dl. One month later, the patient had a second episode of acute respiratory distress and was treated with a short course of cyclophosphamide despite negative assays for anti-GBM-antibodies. There has been no recurrence of symptoms over 10 years of follow-up and the patient currently has a serum-creatinine of 0.58 mg/dl.

**Patient 6** was admitted with pulmonary haemorrhage and respiratory failure requiring mechanical ventilation. Serum-creatinine was 1.83 mg/dl and anti-GBM-antibody titer was 1∶40. A renal biopsy showed a focal necrotizing glomerulonephritis with 19% crescents and linear IgG deposition along the GBM. She was treated with dexamethasone (initially 100 mg over three days), cyclophosphamide (five boluses of 750 mg cyclophosphamide) and 13 sessions of IAS over one month. Respiratory failure resolved rapidly and serum-creatinine, after increasing to a maximum of 3.7 mg/dl on day 3 normalized within a month. For eight years she remained well but then presented with a recurrence of anti-GBM-disease with hemoptysis, dyspnoea and positive anti-GBM-antibodies. The symptoms resolved after a course of six boli of cyclophosphamide and she has since enjoyed excellent renal function.

**Patient 7** presented with acute renal failure and dyspnoea. Serum-creatinine was 14.5 mg/dl and anti-GBM-antibody concentration was 207.8 U/ml. Renal biopsy showed necrotizing glomerulonephritis (100% crescents with linear IgG deposition). Hemodialysis was started and he was treated with pulse methylprednisolone (1 g daily for three doses) followed by tapering doses of prednisolone and oral cyclophosphamide. He received PE twice before institution of 15 IAS sessions over three weeks. Pulmonary disease resolved but the patient remained dialysis-dependent. After eight months the patient received a kidney transplant with a stable serum-creatinine of 1.5 mg/dl and no anti-GBM-antibody recurrence.

**Patient 8** presented with dialysis-dependent acute renal failure, haemoptysis and dyspnoea. Serum-creatinine was 6.1 mg/dl and anti-GBM-antibody concentration was 58.5 U/ml. Renal biopsy showed necrotizing glomerulonephritis (96% crescents and linear IgG deposits). He was treated with three 1 g pulses of methylprednisolone followed by oral prednisolone and cyclophosphamide. He received four PEs but anti-GBM-antibodies remained positive. After conversion to IAS (15 sessions over three weeks) pulmonary haemorrhage resolved swiftly and renal function recovered after 2 weeks with a final serum-creatinine of 2.5 mg/dl. After 10 months serum-creatinine increased to 3.75 mg/dl. He was given a second course of steroids and 17 IAS sessions, although assays for anti-GBM-antibodies were negative; renal biopsy showed severe sclerosis. Two years after presentation he restarted dialysis currently awaiting renal transplantion.

**Patient 9** presented with serum-creatinine of 4.99 mg/dl necessitating hemodialysis. A renal biopsy showed necrotising glomerulonephritis (94% crescents and segmental scars affecting half of the glomeruli and linear IgG deposition along the GBM) and serum anti-GBM-antibodies were 122.6 U/ml. He was treated with prednisolone (100 mg/day tapering), pulse cyclophosphamide and PE. Anti-GBM-antibodies remained positive and his condition deteriorated despite three PE cycles and IAS was substituted (10 sessions over two weeks). He remained on hemodialysis until receiving a renal graft 18 months later. Due to incompliance he required a second allograft but is now well with stable renal function and no recurrence of anti-GBM-disease.

**Patient 10** presented with acute dialysis-dependent renal failure one year after a nephrectomy due to arterial rupture. Anti-GBM-antibody concentration was 51.4 U/ml. Biopsy showed necrotizing glomerulonephritis (63% crescents and linear IgG deposition). The nephrectomy specimen confirmed vasculitis accompanied by a mild focal proliferative glomerulonephritis without detectable IgG deposits, ANCA were negative. She was treated with prednisolone and pulse cyclophosphamide. Anti-GBM-antibodies were reduced by 86% with one IAS treatment and remained negative after 5 treatments. She regained independent renal function after one month and is currently well with a serum-creatinine of 2.96 mg/dl and no detectable anti-GBM-antibodies.

### Anti-GBM-antibody removal

**Figure 1:**
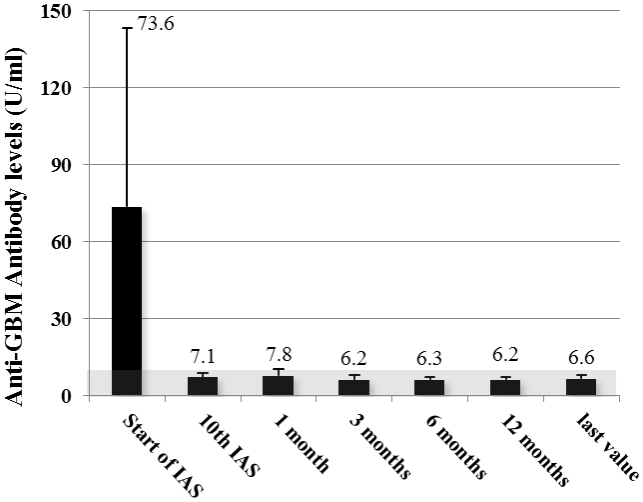
Anti-GBM antibody levels from start of immunoadsorption until end of observation. Mean values ± standard deviation of all patients measured with ELISA depicted. Grey area denotes negativity of the assay.

IAS was highly effective at removing anti-GBM-antibodies with a reduction of 71.1 to 86.4% per treatment, and in controlling anti-GBM-antibody concentrations. Combined with immunosuppression, anti-GBM-antibodies became undetectable (<10 U/ml) in all patients within 2 to 9 IAS treatments (Figure 1). Autoantibody concentrations decreased from a mean of 73.6 U/ml (range 6.6 to 207.1) at IAS start to 7.8 U/ml after one month and remained undetectable thereafter. The mean value was 6.2 U/ml after a year and 6.6 U/ml at latest follow up (mean 29 months after presentation, Table 3). An exception was patient 6 who had a pulmonary relapse of anti-GBM-disease after eight years which resolved quickly with cyclophosphamide but otherwise enjoyed excellent renal function.

**Table 3:** Renal function and patient survival.

Patients	Follow-Up	Patient survival		Independent renal function			
		1 year	Last FU	Start ofIAS	End ofIAS	1 year	Last FU
(n)	mean, range	n (%)	n (%)	n (%)	n (%)	n (%)	n (%)
10	84, 9–186	9 (90%)	9 (90)	4 (40)	7 (70)	5 (63)	5 (50)

**Figure 2:**
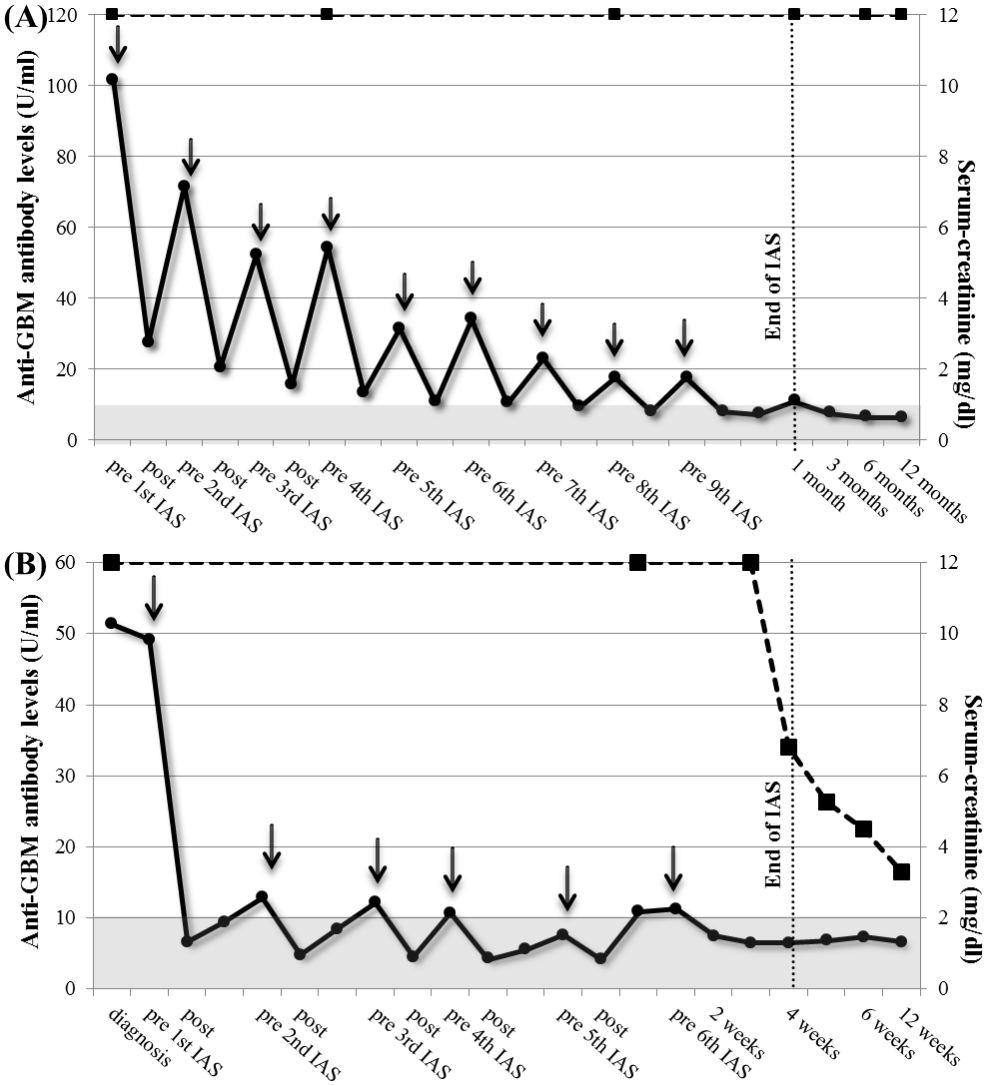
Anti-GBM antibody kinetics and renal function in patient #7 (2a) and patient #10 (2b). Antibody values at diagnosis, before and after IAS, 12 hours in between sessions and up to 6 weeks; arrows depict daily IAS sessions. Grey area depicts negativity of the assay.

We studied the kinetics of anti-GBM-antibody removal and resynthesis in one patient more detail (Figure 2). Anti-GBM-antibody concentrations fell from 49.2 to 6.7 U/ml (86.4%) during the first IAS session and were consistently less than 10 U/ml after 5 IAS sessions. Anti-GBM-antibodies were measured before and after each IAS and at the midpoint between treatments after equilibration between intra- and extravascular was complete. Consequently the difference between the mid-point and pre-IAS values provides an estimate of the rate of anti-GBM-antibody synthesis indicating that anti-GBM-antibody synthesis was substantially reduced after four IAS, being negligible after five. IAS was equally efficient at removing IgG1, IgG2 and IgG4 (85%, 81% and 85% respectively) whereas IgG3 was removed with about half the efficiency (44%, Figure S1).

### Renal function and patient survival

**Figure 3:**
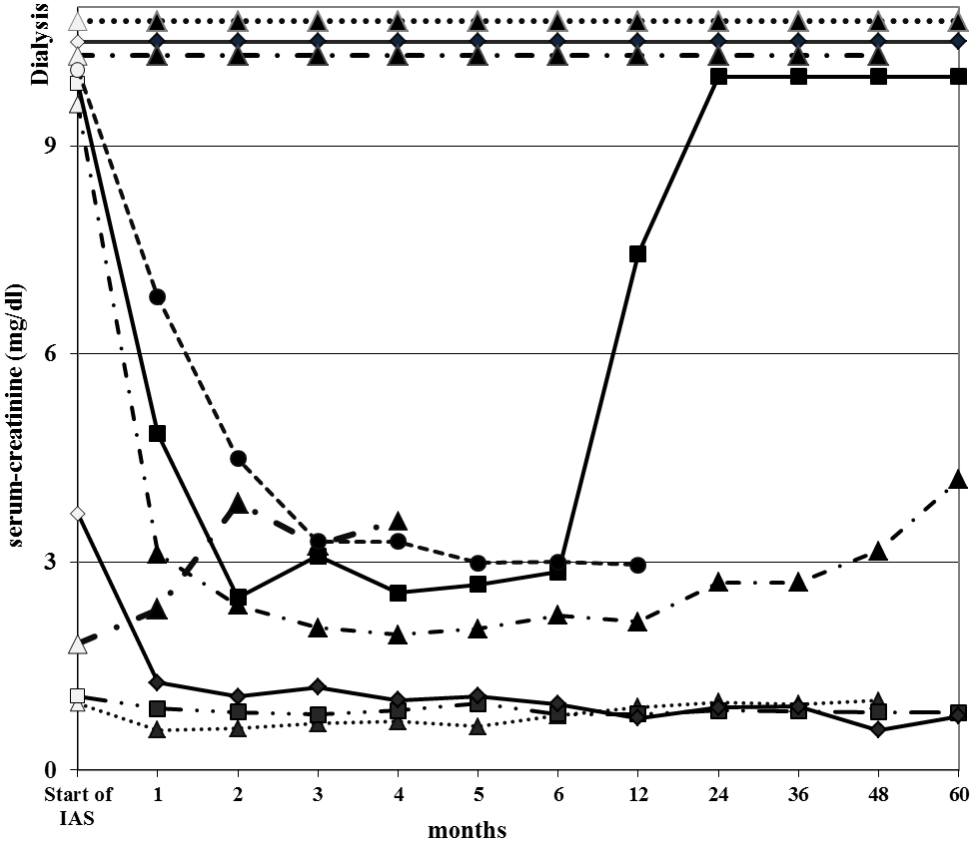
Dynamics of serum-creatinine of individual patients. Observation period from start of IAS until 5 years. White coloured time points depict positive anti-GBM antibody testing.

Pulmonary haemorrhage resolved rapidly after starting immunosuppression and IAS enabling discontinuation of artificial ventilation in the two patients who had previously needed it. Renal function improved in 4 of 8 patients with increased serum-creatinine, including three of six who where initially dialysis dependent with 100%, 96% and 63% crescents on their renal biopsies (Figure 3). Consequently renal survival increased from 40% at presentation to 70% at the end of IAS (mean 3 months) and decreased to 63% after 1 year and 50% at last follow-up (mean 84 months, range 9–186), respectively (Table 3).

One patient died of fungal infection including septic renal failure after control of his anti-GBM-disease whilst the remaining 9 patients were alive at last follow-up (mean 84 months).

### Adverse events

No anaphylactic adverse events were observed during or after IAS. Infections were rare with only one infection requiring hospitalization. There was no reported case of malignancy over a combined follow-up of 70 patient years.

### Cost analysis

**Figure 4:**
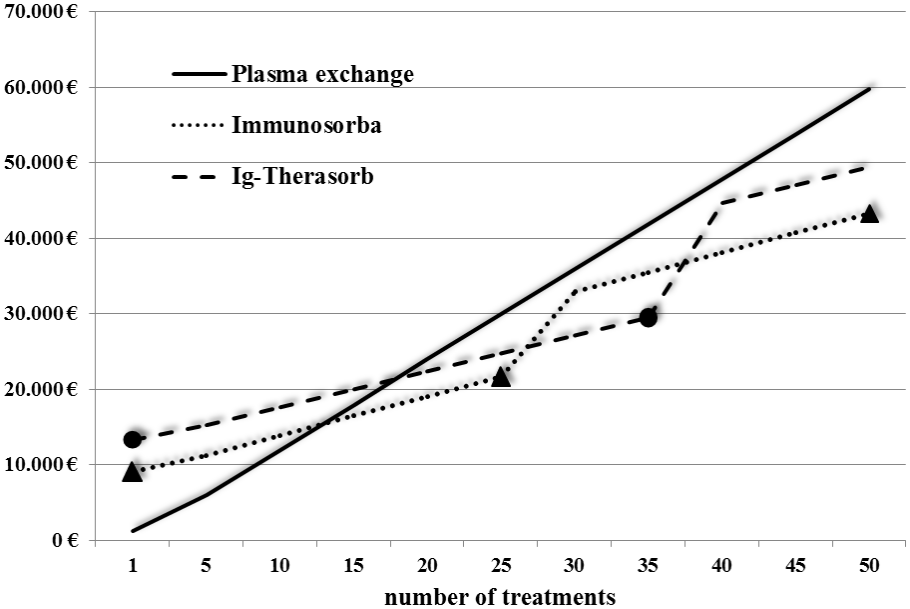
Cumulative costs of immunoadsorption and plasma exchange. Circles and triangles depict earliest timepoints of adsorber change due to reduced adsorption capacity (every 25 treatments for Immunosorba, every 35 treatments for Globaffin).

The per-treatment cost of IAS varies depends on the type of adsorber and on the number of treatments performed as the absorber can be reused. In our study, the mean number of IAS per patient was 23, resulting in the cost of 896 € per single Immunosorba IAS treatment including all disposables and personnel time. The costs for 23 Therasorb treatments were 1035 € per treatment, PE with 4 liters of human albumin including personnel and disposables costs 1194 € at our center (Figure 4).

## Discussion

Our study provides the largest evaluation of a series of patients with anti-GBM-disease treated with IAS whose use has only previously been reported in case studies [21]–[24]. Our results demonstrate that a) the IAS protocol presented enables rapid removal of anti-GBM-antibodies, with efficiency exceeding that of PE; b) that IAS and immunosuppression led to rapid clinical improvement both of pulmonary haemorrhage and of renal function and c) that IAS is an economical choice in anti-GBM-disease. As always in conditions as rare as anti-GBM-disease, the conclusions need to be qualified because of the number of patients studied and retrospective design. Nonetheless, the consistency of the patient responses suggests that IAS is a safe and cost-effective alternative to PE for the management of anti-GBM-disease.

IAS was effective at removing all IgG subclasses and anti-GBM-antibodies were 71 to 84% lower after the first IAS and in normal range after 2 to 9 treatments. Anti-GBM-antibody synthesis may have already ceased by this time as demonstrated in patient 10 in whom we analyzed autoantibody kinetics.

These results are consistent with previous studies by Peters et al. [26] who showed that the combination of anti-GBM-antibody removal with PE and immunosuppression with cyclophosphamide shortened the duration of autoantibody synthesis compared to either treatment. However, both the rapidity of anti-GBM-antibody reduction and duration of synthesis were shorter in our patients treated with IAS than in patients treated with PE [26]. The rate of antibody removal was greater in our patients than in the four patients treated by IAS reported by Hu et al [21] but they processed one plasma volume (3L) at each session whereas we processed 2.5–3 plasma volumes (8L) at each session.

Clinical studies have consistently demonstrated the need for rapid control of anti-GBM-antibody concentration if renal function is to improve and have emphasised the poor prognosis for those with compromised renal function before this is achieved [7], [8], [11], [27]. For example, only two of 39 dialysis-dependent patients treated with PE and cyclophosphamide were alive with renal function after 1 year in the cohort studies by Levy et al., and accordingly Cui et al. reported 63 patients with serum-creatinine ≥6.8 mg/dl of which only 2 were alive and had intact renal function after one year [11]. Against this background, the recovery of renal function in three of the six dialysis dependent patients in our group was unexpected, especially as renal biopsies showed fulminant crescents. Recovered patients remained free of dialysis for 64, 23 and 9 months (still free so far). These excellent results may be attributable to the intensive anti-GBM-antibody removal and more rapid control of circulating autoantibody concentrations achieved by our protocol.

Long-term patient and renal survival in our study were 90% and 50% respectively at the last follow-up (mean of 84 months) compared to 54% and 35% at a median of 90 months (71 patients) reported by Levy et al [8].

Treatment with IAS is commonly considered prohibitively expensive compared to PE but this is not the case for those needing intensive apheresis therapy as is usual in anti-GBM-disease. Costs for IAS gradually decreased due to reusable adsorbers resulting in lower total treatment costs than PE (exchange against 4 liters human albumin 5%) for those receiving 15 or more treatments. Furthermore, IAS-adsorbers can be stored for up to two years and are potentially available to treat early relapses (Table S1).

The safety profile of IAS was highly acceptable and there were no major procedure-related adverse events. One patient died with sepsis after renal function had been improved. The excellent safety profile when used with cyclophosphamide and high dose steroids is consistent with reports in other autoimmune diseases [28], [29].

In conclusion, we have shown that IAS is a highly efficient, safe and cost-effective technique to remove autoantibodies in anti-GBM-disease compared to PE. We also demonstrate the feasibility of increasing the volume of plasma processed at each session from one plasma volume as is normal with PE to 3 plasma volumes. With this regimen, anti-GBM-antibodies consistently became undetectable more rapidly than has been reported with PE, which could translate into a major clinical benefit. Further studies are necessary to determine the clinical value of IAS as potential standard management of anti-GBM disease.

## Supporting Information

Figure S1 **Reduction of IgG subclasses through IAS in patient #10.** Antibody values at diagnosis, before and after IAS, 12 hours in between sessions for 5 treatments. Arrows depict daily IAS sessions.
(TIF)

Table S1 **Cost analysis of Immunoadsorption and Plasma exchange.**
(DOCX)
