# Supplementary material for: Long-Term Outcome of Anti-Glomerular Basement Membrane Antibody Disease Treated with Immunoadsorption
Source: PLoS One. 2014 Jul 31;9(7):e103568. doi: 10.1371/journal.pone.0103568 (PMC4117516; doi:10.1371/journal.pone.0103568)
Supplement: Table S1 — Cost analysis of Immunoadsorption and Plasma exchange. (DOCX) [file pone.0103568.s002.docx]

***Supplementary Table 1.* Cost analysis of Immunoadsorption and Plasma exchange.**

| **Immunoadsorption** | | | **Required for 1 treatment** | |
| --- | --- | --- | --- | --- |
| **Product/Personnel** | **Costs (€)** | **Items per Set** | **Ig-Therasorb** | **Protein A** |
| Immunosorba® Adsorber | 8600 | 2 |  | 2 |
| Prot A ADA Set | 625 | 10 |  | 1 |
| Therasorb® Adsorbers | 12800 | 2 | 2 |  |
| Therasorb® ADA Set | 390 | 10 | 1 |  |
| PlasmaEx Set | 951,6 | 6 | 1 | 1 |
| AdapterSet ADA/PE | 126,84 | 12 | 1 | 1 |
| Immunosorb® Preserv | 150 | 10 |  | 1 |
| PBS + 0.01% NA-AZID | 56,68 | 6 | 1 |  |
| Nurse | 186 | Not applicable | 1 | 1 |
| Doctor | 74,4 | Not applicable | 1 | 1 |
| **Total costs for 20 treatments** | | | 22.360,33 | 19.041,40 |
| **Costs per treatment when 20 times performed** | | | **1.118,02** | **952,07** |
|  | | | | |
| **Plasma exchange** | | | **Required for 1 treatment** | |
| **Product/Personnel** | **Costs (€)** | **Items per Set** | **with FFP** | **with HA 5%** |
| Set Apherese PL1 | 171,38 | 1 | 1 | 1 |
| Set HPFß617 | 22,04 | 1 | 1 | 1 |
| HA 5% 500 ml | 98,7 | 1 | 0 | 8 |
| 1 Unit of FFP | 32,97 | 1 | 20 | 0 |
| Nurse | 124 | Not applicable | 1 | 1 |
| Doctor | 74,4 | Not applicable | 1 | 1 |
| MTA | 13 | Not applicable | 1 | 1 |
| **Total costs for 20 treatments** | | | 21284,4 | 23888,4 |
| **Costs per treatment when 20 times performed** | | | **1064,22** | **1194,42** |
